# Effect of Polyethylene Glycol Modification of TiO2 Nanoparticles on Cytotoxicity and Gene Expressions in Human Cell Lines

**DOI:** 10.3390/ijms13033703

**Published:** 2012-03-21

**Authors:** Sharmy Saimon Mano, Koki Kanehira, Shuji Sonezaki, Akiyoshi Taniguchi

**Affiliations:** 1 Cell-Materials Interaction Group, Biomaterials Unit, Nano-Bio Field, International Center for Materials Nanoarchitectonics (MANA), National Institute for Materials Science, 1-1, Namiki, Tsukuba, Ibaraki 305-0044, Japan; E-Mail: saimon.sharmy@nims.go.jp; 2 Graduate School of Advanced Science and Engineering, Waseda University, 3-4-1 Okubo, Shinjuku, Tokyo 169-8555, Japan; 3 TOTO Ltd. Research Institute, Nakashima 2-1-1, Kokurakita, Kitakyushu 802-8601, Japan; E-Mails: koki.kanehira@toto.co.jp (K.K.); shuji.sonezaki@jp.toto.com (S.S.)

**Keywords:** nanoparticles, titanium dioxide, nanotoxicology, gene expression

## Abstract

Nanoparticles (NPs) are tiny materials used in a wide range of industrial and medical applications. Titanium dioxide (TiO_2_) is a type of nanoparticle that is widely used in paints, pigments, and cosmetics; however, little is known about the impact of TiO_2_ on human health and the environment. Therefore, considerable research has focused on characterizing the potential toxicity of nanoparticles such as TiO_2_ and on understanding the mechanism of TiO_2_ NP-induced nanotoxicity through the evaluation of biomarkers. Uncoated TiO_2_ NPs tend to aggregate in aqueous media, and these aggregates decrease cell viability and induce expression of stress-related genes, such as those encoding interleukin-6 (IL-6) and heat shock protein 70B’ (HSP70B’), indicating that TiO_2_ NPs induce inflammatory and heat shock responses. In order to reduce their toxicity, we conjugated TiO_2_ NPs with polyethylene glycol (PEG) to eliminate aggregation. Our findings indicate that modifying TiO_2_ NPs with PEG reduces their cytotoxicity and reduces the induction of stress-related genes. Our results also suggest that TiO_2_ NP-induced effects on cytotoxicity and gene expression vary depending upon the cell type and surface modification.

## 1. Introduction

Nanoparticles (NPs) are tiny materials (diameter of 1 to 100 nm in at least one dimension) [1] characterized by a very high surface area-to-volume ratio [2]. Due to the unique properties afforded by their size, NPs possess a wide range of applications in the industrial, electrical, agricultural, pharmaceutical, and medical fields. However, despite the wide application of nanomaterials, little is known about their impact on human health and the environment. As a result, considerable effort has been expended on identifying the potential toxicity of NPs to cells and organisms. It has been suggested that the small size and corresponding high specific surface area are the major determinants of NP toxicity [3]. It has also been proposed that the surface area of NPs greatly increases their ability to produce potentially toxic reactive oxygen species (ROS) [4].

Titanium dioxide (TiO_2_), which is used as a photocatalyst [5] in air and water cleaning and is found in a wide array of products including paints, pigments, cosmetics, and skin care products [6], has been classified as a biologically inert substance with respect to both animals and humans [7,8]. TiO_2_ NPs can be applied in biomedical fields, for example, TiO_2_ NPs could be used for sonodynamic therapy for cancer [9]. However, recent investigations revealed that rats exposed to ultra fine TiO_2_ (UF-TiO_2_) NPs develop inflammation, pulmonary damage, and lung tumors [10,11]. This toxicity may be due to the ease with which these NPs can pass through the cell membrane and disrupt biological systems [12]. The design and evaluation of proper safety measures for NPs thus necessitates a more complete understanding of how nanomaterials interact with cells.

Polyethylene glycol (PEG) is a coiled polymer of repeating ethylene ether units with a dynamic conformation. PEG is inexpensive, versatile, and FDA-approved for many applications [13]. In addition, PEG is non-toxic and non-immunogenic, and has favorable pharmacokinetics and tissue distribution [14]. Modifying the surface of NPs with PEG (PEGylation) not only prevents agglomeration [15], but also renders NPs resistant to protein adsorption and enhances their biocompatibility [16]. Coating nanomaterials with PEG also increases the *in vivo* circulation time, thereby likely reducing clearance via the reticuloendothelial system (RES) [17]. PEGylated single walled carbon nanotubes exhibited less cytotoxic potency than uncoated ones [18].

Studies of the effects of TiO_2_ NPs in rodent lungs have shown that NPs induce elevated expression of proinflammatory factors such as interleukins 1 (IL-1) and 6 (IL-6), tumor necrosis factor-α (TNF-α), macrophage inhibitory protein, and monocyte chemotactic protein [19]. In our previous study [20], we examined the cytotoxicity of two types of TiO_2_ NP aggregates: small-TiO_2_ NPs (166 nm) and large-TiO_2_ NPs (596 nm). Cytotoxicity and mRNA expression analyses indicated that large-TiO_2_ NP aggregates have a greater effect on cell viability and the expression of molecular marker genes, such as heat shock protein (HSP) and IL-6, than do the small-TiO_2_ aggregates using NCI-H292 and THP-1 cells. We also developed a sensor cell for evaluating nanomaterial biosafety that assesses NF-κB pathway activation to detect TiO_2_ NP-induced inflammation [21].

Here, we report the results of experiments aimed at reducing the cytotoxicity and induction of gene expression associated with TiO_2_ NP exposure by modifying the surface of TiO_2_ NPs with PEG. This study focused on the effects of PEG-conjugated TiO_2_ (PEG-TiO_2_-49.6 nm) at the cellular and gene expression levels. We conducted cell viability testing and mRNA expression analysis in different cell lines to assess how PEG modification affects stress and toxicity. Our results indicate that modification of TiO_2_ NPs with PEG reduces both their cytotoxicity and the induction of toxicity marker gene expression.

## 2. Results and Discussion

### 2.1. Viability of Cells Exposed to PEG-TiO_2_ NPs

In our previous study, we demonstrated the effects of exposure to TiO_2_ NP aggregates on cell viability using two different human cell lines [20]. The results indicated that high concentrations of TiO_2_ NP aggregates have a negative impact on cell viability. NCI-H292 cells exposed to 20 μg/mL of TiO_2_ NP aggregates showed about 80% viability [20]. In this study, we focused on the effects of PEG-TiO_2_ NPs, which we predicted would be less toxic and induce less expression of genes associated with stress and toxicity. Since it is not clear how NPs affect different cell types, we utilized four different human cell lines in this study.

**Figure 1:**
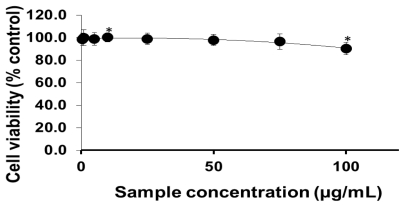
Cell viability testing based on cytoplasmic ATP concentration. NCI-H292 cells were exposed to the indicated concentrations of PEG-TiO_2_ NPs for 24 h. Results are shown as the mean ± SD, *n* ≥ 3 for each concentration. * *P* < 0.01.

**Figure 2:**
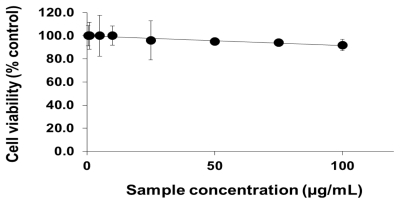
Cell viability testing based on cytoplasmic ATP concentration. THP-1 cells were exposed to the indicated concentrations of PEG-TiO_2_ NPs for 24 h. Results are shown as the mean ± SD, *n* ≥ 3 for each concentration.

**Figure 3:**
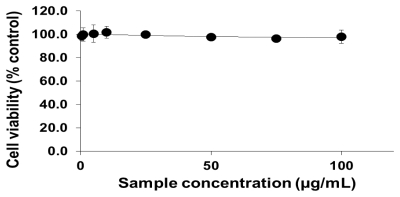
Cell viability testing based on cytoplasmic ATP concentration. HeLa cells were exposed to the indicated concentrations of PEG-TiO_2_ NPs for 24 h. Results are shown as the mean ± SD, *n* ≥ 3 for each concentration.

**Figure 4:**
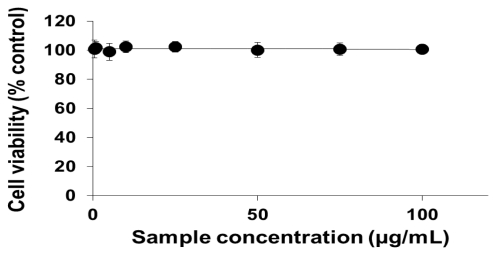
Cell viability testing based on cytoplasmic ATP concentration. HepG2 cells were exposed to the indicated concentrations of PEG-TiO_2_ NPs for 24 h. Results are shown as the mean ± SD, *n* ≥ 3 for each concentration.

To analyze the cellular effects of PEG-TiO_2_, different cell lines (NCI-H292, THP-1, HeLa and HepG2) were exposed to NPs. Cells with no exposure to NPs were also tested as controls for those cell lines. To determine the effect of PEG-TiO_2_ NP exposure on cell viability, the concentration of cytoplasmic ATP (which signals the presence of metabolically active cells) was determined after 24 h of exposure. At a high concentration of PEG-TiO_2_ NPs, the viability of both NCI-H292 and THP-1 cells decreased slightly to 95% (Figures 1 and 2, respectively). There was no apparent change in the viability of HeLa and HepG2 cells after 24 h of exposure to PEG-TiO_2_ NPs (Figures 3 and 4). In a similar experiment involving 6 h of exposure to PEG-TiO_2_ NPs, all cell lines maintained 100% viability (data not shown). Our findings thus indicate that modification of TiO_2_ NPs with PEG reduces the cytotoxicity of the particles. In addition, our data indicate that the cytotoxicity of PEG-TiO_2_ NPs differs between cell lines.

### 2.2. mRNA Expression Analysis of NP-Exposed Cells

To identify potential biomarkers of nanoparticle toxicity, we conducted a Human Stress and Toxicity Pathway Finder PCR array analysis of 84 genes indicative of stress and toxicity, using NCI-H292 cells exposed to PEG-TiO_2_ NPs for 6 h. Genes selected as biomarker candidates based on the PCR array results included CCNG1, CRYAB, CSF-2, CYP2E1, CYP7A1, FMO1, FMO5, GSTM3, HMOX-1, HSPA6, IL-6, LTA, TNF, and UGT1A4 (Table 1).

**Figure 5:**
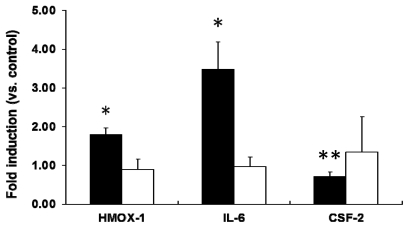
Expression of stress and toxicity marker mRNAs in PEG-TiO_2_ NP-exposed NCI-H292 cells. Cells were exposed for 6 h (solid black bars) or 24 h (open bars). Results are shown as the mean ± SD, *n* ≥ 3 for each marker. * *P* < 0.05, ** *P* < 0.01.

We next investigated the level of mRNA expression of stress- and toxicity-associated molecular markers in PEG-TiO_2_ NP-exposed cells using RT-PCR. Selected molecular markers included the oxidative marker HMOX-1 and the inflammation markers IL-6 and CSF-2, because expression of mRNAs for these markers was statistically higher than the other markers after treatment with PEG-TiO_2_ NPs. In PEG-TiO_2_ NP-exposed NCI-H292 cells, a significant increase in the level of IL-6 mRNA was observed after 6 h (Figure 5, solid black bars). The level of HMOX-1 mRNA was also significantly increased (2-fold higher than in control cells); however, the level of CSF-2 mRNA was lower than that in the control cells after 6 h of exposure. There was no significant difference in the level of HMOX-1, IL-6, or CSF-2 mRNA expression when the exposure duration was increased to 24 h (Figure 5, open bars).

**Figure 6:**
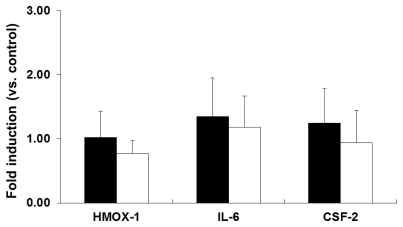
Expression of stress and toxicity marker mRNAs in PEG-TiO_2_ NP-exposed THP-1 cells. Cells were exposed for 6 h (solid black bars) or 24 h (open bars). Results are shown as the mean ± SD, *n* ≥ 3 for each marker.

In the case of THP-1 cells, no significant changes in the induction of HMOX-1, IL-6, or CSF-2 mRNA expression were observed after 6 h (Figure 6, solid black) or 24 h (Figure 6, open bars) of exposure to PEG-TiO_2_ NPs. These results indicate that PEG-TiO_2_ NPs do not induce expression of these molecular markers in THP-1 cells.

**Figure 7:**
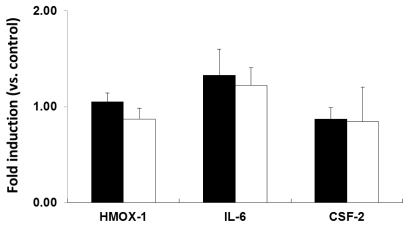
Expression of stress and toxicity marker mRNAs in PEG-TiO_2_ NP-exposed HeLa cells. Cells were exposed for 6 h (solid black bars) or 24 h (open bars). Results are shown as the mean ± SD, *n* ≥ 3 for each marker.

**Figure 8:**
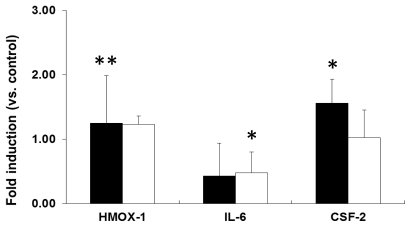
Expression of stress and toxicity marker mRNAs in PEG-TiO_2_ NP-exposed HepG2 cells. Cells were exposed for 6 h (solid black bars) or 24 h (open bars). Results are shown as the mean ± SD, *n* ≥ 3 for each marker. * *P* < 0.05, ** *P* < 0.01.

Similarly, neither 6 h nor 24 h of exposure to PEG-TiO_2_ NPs resulted in induction of HMOX-1, IL-6, or CSF-2 mRNA expression in HeLa cells (Figure 7). In HepG2 cells, 6 h of PEG-TiO_2_ NP exposure resulted in significant induction of CSF-2 mRNA expression compared to control cells (Figure 8, solid black bars), and a reduction in IL-6 mRNA expression after 24 h of exposure (Figure 8, open bars). The data indicate that each cell line responds differently to PEG-TiO_2_ NP exposure, which suggests that the induction of mRNA expression depends upon both the NPs and the cell type.

In our previous study, we demonstrated that exposure to high concentrations of TiO_2_ NP aggregates affects the expression of a number of genes [20]. In NCI-H292 cells, expression of the genes encoding heat shock protein 70B’ (HSP70B’) and IL-6 increased 100-fold and 10-fold, respectively, upon exposure to TiO_2_ NP aggregates. Exposure to TiO_2_ NP aggregates resulted in a six-fold increase in expression of the IL-6 gene in THP-1 cells [20]. In the present study, no induction of HSP70B’ expression upon PEG-TiO_2_ NP exposure was observed in any of the cell lines examined (data not shown). An induction of only approximately 3.5-fold was observed in the NCI-H292 cells exposed to PEG-TiO_2_ NPs. Our results thus indicate that modifying the surface of TiO_2_ NPs with PEG reduces the induction of genes associated with stress and toxicity.

### 2.3. Discussion

Titanium dioxide has a wide range of applications. Uncoated TiO_2_ NPs, like other insoluble nanomaterials, tend to clump together in aqueous media [22]. In our previous study [20], we focused on the effect of TiO_2_ NP aggregates on cytotoxicity and gene expression. A series of *in vivo* and *in vitro* studies [23–28] of aggregated TiO_2_ NPs showed that these particles stimulate the production of ROS and induce an inflammatory response that damages the cells and neurons and affects the central nervous system. In order to reduce these deleterious biological effects of TiO_2_ NPs, we conjugated TiO_2_ NPs with PEG to minimize the formation of aggregates. PEG possesses a number of important physicochemical and biological properties, including hydrophilicity, solubility in water and organic solvents, and lack of toxicity [29,30]. The present study focused primarily on the effects of PEG-TiO_2_ NPs in different cell lines. The number of viable NCI-H292 cells decreased slightly upon exposure to high concentrations of PEG-TiO_2_ NPs, indicating that PEG-TiO_2_ exposure results in a minimal degree of stress in these cells. In our previous study, we demonstrated that exposure to large-TiO_2_ NP aggregates reduces the number of viable NCI-H292 cells by around 20%, as compared to exposure to small-TiO_2_ NP aggregates. As demonstrated here, treatment with PEG-TiO_2_ NPs reduces the number of viable NCI-H292 cells by only around 5%, as compared to TiO_2_ NPs (Figure 1). Our results thus indicate that modification with PEG reduces the cytotoxic effects of TiO_2_ NPs. However, it is difficult to distinguish between surface characters and cytotoxic activities, because PEG coated TiO_2_ NPs changed not only surface character, but also particle aggregate size.

We also performed PCR array analysis of 84 genes that are indicative of stress and toxicity. The mRNA expression analysis was performed, based upon the data obtained from the PCR array. PCR array is a sensitive technique used to screen for biomarkers related to human stress and toxicity. In our experiments, gene expression in response to PEG-TiO_2_ NP exposure varied with cell type. NCI-H292 cells responded quickly to PEG-TiO_2_ NP exposure (6 h) with induction of the proinflammatory cytokine IL-6 and oxidative stress marker HMOX-1, and a significant reduction in expression of the cytokine CSF-2. However, the pattern of gene expression changed when the duration of NP exposure was increased to 24 h, probably because the cells had adapted to the presence of NPs, and therefore did not show induction of any of the biomarkers. The same experiment was conducted using THP-1, HeLa, and HepG2 cells; however, the response of these cell lines differed from that of the NCI-H292 cell line. Induction of stress and toxicity biomarkers upon PEG-TiO_2_ NP exposure was observed only in NCI-H292 cells. In HepG2 cells especially, PEG-TiO_2_ NP reduced proinflammatory cytokine IL-6, but induced anti-inflammatory CSF-2. Some studies have shown that increased levels of proinflammatory cytokines such as TNF-α, IL-1, and IL-6, coupled with the oxidative stress resulting from increased generation of ROS, induces cardiac dysfunction [31,32]. However, another study showed that inhalation of TiO_2_ gives rise to a more pronounced inflammatory response than does inhalation of the same mass of larger TiO_2_ particles [33].

One mechanism whereby NPs can be transported into cells is through a process called endocytosis [34]. Endocytosis involves multiple processes that fall into two categories: phagocytosis and pinocytosis. Phagocytosis is a principal component of the body’s innate immunity in which macrophages internalize targets in an actin-dependent manner. Thus, phagocytosis by macrophages is critical for the uptake of large particles (0.2–10 μm) [35]. Monocytes/macrophages and neutrophils have been described as professional phagocytes [36]. Pinocytosis occurs in all cell types and is mediated by at least four basic mechanisms: macropinocytosis, clathrin-mediated endocytosis, caveolae-mediated endocytosis, and clathrin-caveolae independent endocytosis [37]. Macropinocytosis is the non-selective endocytosis of solute molecules [38], and is associated with the formation of actin-dependent membrane ruffles up to 5 μm in diameter [39]. Clathrin-mediated endocytosis is the most important mechanism for receptor-mediated endocytosis, and involves the formation of clathrin-coated pits that are 100 to 200 nm in size [40]. Caveolae are 50- to 80-nm flask-shaped plasma membrane invaginations found in many cell types and are marked by the presence of a member of the caveolin protein family [41]. The mechanisms that govern clathrin-caveolae independent endocytosis remain poorly understood. It is assumed that THP-1 cells take up NPs via phagocytosis. However, the entry of NPs into the cells depends upon the size and surface properties of the NPs and the presence of appropriate cell membrane receptors. Thus, one possible reason for variable responses to NP exposure may be the differences in the uptake mechanism between cell types.

## 3. Experimental Section

### 3.1. Preparation of PEG-TiO_2_

TiO_2_ NPs (Degussa Aeroxide P25: The particle size is 25 nm; The crystal rate of anatase and rutile of P25 is 80% anatase and 20% rutile.) was used for PEG modification. PEG-TiO_2_ was prepared as described previously [9]. The pH of comb-shaped PEG-maleic acid anhydride (PEGMA, AM1510K; Nihon Yushi Co., Ltd., Tokyo, Japan) in water was adjusted to 4.0 using 0.1 M NaOH. To activate the PEGMA carboxyl groups, 0.6 mL of 0.8 M 1-ethyl-3-[3-dimethylaminopropyl] carbodiimide hydrochloride (Pierce, Rockford, IL, USA) was added and the solution was incubated at room temperature for 5 min. Next, 0.3 mL of 0.2 M 4-amino-salicylic acid (4ASA; Wako Jyunyaku, Osaka, Japan) was added and the solution was incubated at 40 °C for 16 h. Unreacted 4ASA and other small molecules were removed by centrifuging five times using Amicon Ultra-15 (MWCO = 3000; Millipore, Billerica, MA, USA) ultra-filters, according to the manufacturer’s instructions. Exchange of the solvent of reacted PEGMA-4ASA was carried out after vacuum drying at 25 °C and 20 hPa for 3 min, and 5 hPa for 35 min. The concentration of PEGMA-4ASA was adjusted to 50 mg/mL using dimethylformamide (DMF). An acidic TiO_2_ solution was prepared using thermal synthesis based on hydrolysis of an organo-titanium compound followed by peptization. After hydrolysis of chlorotitanium triisopropoxide (Acros, Morris Plains, NJ, USA), peptization with HNO_3_ was carried out at 80 °C. The reactant was adjusted to a solid content of 20% (w/v) using 1.5 M HNO_3_. After ultrasonication at 200 kHz for 30 min (Midsonic 600; Kaijyo, Tokyo, Japan), particle-size distribution analysis was conducted, confirming the presence of TiO_2_ particles with a diameter of approximately 40 nm in the solution. The TiO_2_ solution (0.75 mL) was added to 10 mL of DMF, and 5 mL of 50 mg/mL PEGMA-4ASA in DMF was subsequently added, followed by stirring. The solution was incubated at 130 °C for 16 h using Chemist Plaza (Shibata Kagaku, Tokyo, Japan). The reaction was carried out under reflux and vigorous stirring at 600 rpm. After the reaction ended, the solution was cooled to room temperature. Exchange of the solvent of reacted PEG-TiO_2_ was carried out after vacuum drying at 40 °C and 5 hPa for 10 min. The concentration of PEG-TiO_2_ was adjusted to 10 mg/mL using water. The aqueous PEG-TiO_2_ solution was purified by centrifuging five times using Amicon Ultra-15 (MWCO = 3000; Millipore) ultra-filters according to the manufacturer’s instructions. The zeta potential and particle-size distributions of PEG-TiO_2_ were 0.196 mV and 49.6 nm, respectively, measured using a Zetasizer nanoZS (Malvern Instruments, UK), according to the procedures recommended by the manufacturer.

### 3.2. Cell Culture

Human NCI-H292 pulmonary epithelial cells [42] and human THP-1 acute monocytic leukemia cells [43,44] were cultured in RPMI 1640 medium (Invitrogen, NY, USA). Human HeLa cervical cancer cells [45] were cultured in Minimum Essential Medium (MEM) (Invitrogen), and human HepG2 hepatocarcinoma cells [46,47] were maintained in Dulbecco’s Modified Eagle’s Medium (DMEM) (Invitrogen). All media were supplemented with 10% fetal bovine serum (Biowest, UK), 100 U/mL penicillin, and 100 μg/mL streptomycin (Nacalai Tesque, Japan). All cells were maintained under 5% CO_2_ and 100% humidity at 37 °C and cultured in the dark to avoid activation of the titanium surfaces. THP-1 cells were treated with 200 nM phorbol 12-myristate 13-acetate (PMA) (Wako Jyunyaku) for 48 h, after which the old medium was replaced with new medium and the cells were exposed to NPs for either 6 h or 24 h. Cultures of NCI-H292, HeLa, and HepG2 cells that had been seeded 24 h prior were exposed to NPs for 6 h or 24 h.

### 3.3. Cell Viability Test

Cell viability was measured using a Cell Titer-Glo Luminescent Cell Viability Assay kit (Promega, WI, USA) according to the manufacturer’s instructions [48]. For the ATP assay, 1.0 × 10^4^ NCI-H292 cells, 5.0 × 10^4^ THP-1 cells, 2.0 × 10^4^ HeLa cells, and 1.0 × 10^4^ HepG2 cells were seeded in each well of a white opaque-walled 96-well cell culture plate (Nunclone, Roskilde, Denmark). After the cells attained 80% confluence, they were treated with different concentrations of a suspension of PEG-TiO_2_, ranging from 0.00005% (w/v) (0.5 μg/mL) to 0.01% (w/v) (100 μg/mL) for 24 h. Each PEG-TiO_2_ particle suspension was added to the cell culture medium at a volume ratio of 5:100 and cultured for 24 h. The cytoplasmic ATP concentration was then analyzed using a Luminescent cell viability assay reader (Wako Jyunyaku).

### 3.4. Gene Expression Analysis

#### 3.4.1. PCR Array

The PCR array is the most sensitive and reliable method of gene expression analysis. For PCR array analysis, 1.3 × 10^5^ NCI-H292 cells/cm^2^ were seeded in a culture dish and exposed to a suspension of PEG-TiO_2_ NPs at a final concentration of 0.0075% (w/v) (75 μg/mL). Following 6 h of exposure to the PEG-TiO_2_ particles, the cells were detached by mechanical dissociation and the expression of 84 genes that are indicative of stress and toxicity was examined using PCR array analysis as follows. Total cellular RNA was extracted from PEG-TiO_2_-exposed cells using an RNeasy Kit (Qiagen) according to the manufacturer’s instructions. Extracted RNA was treated with DNaseI (Takara), and 4 μg of total RNA was reverse-transcribed into cDNA with a random hexamer primer using the PrimeScript II 1st strand cDNA Synthesis System (Takara) according to the manufacturer’s protocol. PCR array analysis was performed using an ABI PRISM 7000 Sequence Detection System (Applied Biosystems, Singapore). A standard reaction was prepared in 96-well plates (SA Bioscience, Qiagen) containing forward and reverse primers for several genes indicative of stress and toxicity. The cDNA was diluted five-fold with distilled water and mixed with 2× PCR master mix according to the manufacturer’s protocol. An aliquot of reaction mixture (25 μL) containing the test cDNA was then added to each well. The thermocycling conditions were 95 °C for 10 s followed by 40 cycles of 95 °C for 15 s and 60 °C for 1 min. A control reaction was also performed using cDNA prepared from cells that were not exposed to NPs.

#### 3.4.2. Real-Time (RT) PCR

For mRNA expression analysis, 1.3 × 10^5^ NCI-H292 cells/cm^2^, 1.9 × 10^5^ THP-1 cells/cm^2^, 2.2 × 10^5^ HeLa cells/cm^2^, and 1.1 × 10^5^ Hep G2 cells/cm^2^ were seeded in cell culture dishes, and the cells were then exposed to a suspension of PEG-TiO_2_ NPs at a final concentration of 0.0075% (w/v) (75 μg/mL) for 6 h or 24 h. Following exposure, cells were detached by mechanical dissociation and subjected to gene expression analysis. The expression of marker genes was determined using quantitative real-time PCR (RT-PCR) as follows. Total cellular RNA was extracted from titania-exposed cells using an RNeasy Kit (Qiagen) according to the manufacturer’s instructions. Extracted RNA was treated with DNaseI (Takara), and 2 μg of total RNA was reverse-transcribed into cDNA with a random hexamer primer using the PrimeScript II 1st strand cDNA Synthesis System (Takara) according to the manufacturer’s protocol. Real-time PCR was performed using an ABI PRISM 7000 Sequence Detection System (Applied Biosystems). A standard reaction was prepared in 96-well plates (Micro Amp, Applied Biosystems). The reaction mixture was composed of 10 μL of SYBR Premix Ex Taq II (Takara), 10 pmol each of the forward and reverse primers, 2 μL of cDNA, and distilled water to a final volume of 20 μL. The thermocycling conditions were 95 °C for 30 s, followed by 40 cycles of 95 °C for 5 s and 60 °C for 34 s. Normalization of the data was performed using the housekeeping gene glyceraldehyde-3-phosphate dehydrogenase (GAPDH) as an endogenous control in the same reaction as the gene of interest [49]. The primers used in this study were as follows: for GAPDH, forward primer 5′-CCCCCACCACACTGAATCTC-3′ and reverse primer 5′-GCCCCTCCCCTCTTCAAG-3′; for heme oxygenase 1 (HMOX-1), forward primer 5′-GGGTGATAGAAGAGGCCAAGA-3′ and reverse primer 5′-AGCTCCTGCAACTCCTCAAA-3′; for IL-6, forward primer 5′-TGAGTACAAAAGTCCTGA-3′ and reverse primer 5′-TCTGTGCCT GCAGCTTCGT-3′; for granulocyte-macrophage colony stimulating factor 2 (CSF-2), forward primer 5′-TCTCAGAAATGTTTGACCTCCA-3′ and reverse primer 5′-GCCCTTGAGC TTGGTGAG-3′.

### 3.5. Statistical Analysis

Data were expressed as the mean ± SD, (*n* ≥ 3). The data were analyzed using Student *t* test to evaluate the significance of differences between the treated groups and control groups.

## 4. Conclusions

Our previous results indicated that TiO_2_ NPs induce inflammatory and heat shock responses in cells. In order to minimize these responses, we conjugated TiO_2_ NPs with PEG to prevent aggregation. The cytotoxic effects of PEG-TiO_2_ NPs on NCI-H292 cells were less severe than those produced by TiO_2_ aggregates. However, PEG-TiO_2_ NPs did not produce any cytotoxicity in THP-1, HeLa, or HepG2 cells. Analysis of mRNA expression indicated that the expression of particular biomarkers depends upon the cell type. Our results showed that modification of TiO_2_ NPs with PEG reduces their cytotoxicity and reduces the induction of genes associated with stress and toxicity.

## Figures and Tables

**Table 1:** Lists for genes showed induction of mRNA expression in PEG-TiO_2_ particles-exposed NCI-H292 cells after 6 h in PCR array.

Symbols of Genes	Description of the Genes
CCNG1	Cyclin G1
CRYAB	Crystallin, alpha B
CSF2	Colony stimulating factor 2 (granulocyte- macrophage)
CYP2E1	Cytochrome P450, family 2, subfamily E, polypeptide 1
CYP7A1	Cytochrome P450, family 7, subfamily A, polypeptide 1
FMO1	Flavin containing monooxygenase 1
FMO5	Flavin containing monooxygenase 5
GSTM3	Glutathione transferase M3
HMOX1	Heme oxygenase 1
HSPA6	Heat Shock 70kDa protein 6 (HSP70B’)
IL6	Interleukin 6 (interferon, beta 2)
LTA	Lymphotoxin alpha (TNF superfamily, member 1)
TNF	Tumor necrosis factor (TNF superfamily, member 2)
UGT1A4	UDP glucuronosyltransferase 1 family, polypeptide A4
